# Supplementary material for: Transformation of Gardnerella vaginalis with a Bifidobacterium-Escherichia coli shuttle vector plasmid
Source: Microbiol Spectr. 2025 Apr 10;13(5):e00481-25. doi: 10.1128/spectrum.00481-25 (PMC12054147; doi:10.1128/spectrum.00481-25)
Supplement: Supplemental material — Table S1, Figure S1, and Figure S2. [file spectrum.00481-25-s0001.docx]

**Supplemental Table 1:** Results of transformation experiments in which various combinations of competent cell preparation, plasmid amount, electroporation parameters and recovery time were investigated.

| Competent cell prep | Plasmid (ng) | Recovery time (h) | Electroporation parameters | Viability after electroporation | No. of cfu on selective agar |
| --- | --- | --- | --- | --- | --- |
| 10% Glycerol and water | 80 | 2 ½ | 1.5 kV, 400 Ω, 25μF | Yes | 0 |
| 10% Glycerol and water | 100 | 1 ½ | 1.8 kV, 400 Ω, 25μF | Yes | 0 |
| 10% Glycerol and water | 80 | 1 ½ | 1.8 kV, 400 Ω, 25μF | Yes | 0 |
| 10% Glycerol and water | 80 | 2 h | 1.8 kV, 400 Ω, 25μF | Yes | 0 |
| 10% Glycerol and water | 100 | 1 ½ | 1.8 kV, 400 Ω, 25μF | Yes | 0 |
| 10% Glycerol and water | 140 | 1 ½ | 1.8 kV, 600 Ω, 25μF | Yes | 0 |
| 10% Glycerol and water | 60 | 4 | 1.8 kV, 200 Ω, 25μF | Yes | 0 |
| 10% Glycerol and water | 80 | 1 ½ | 2.5 kV, 200 Ω, 25μF | Yes | 0 |
| 10% Glycerol and water | 140 | 4h | 1.8 kV, 600 Ω, 25μF | Yes | 0 |
| 10% Glycerol and water | 600 | 4h | 2.0 kV, 600 Ω, 25μF | Yes | 0 |
| Sucrose Citrate Buffer | 600 | 2h | 1.8 kV, 400 Ω, 25μF | Yes | 0 |
| Sucrose Citrate Buffer | 600 | 4h | 1.8 kV, 400 Ω, 25μF | Yes | 0 |
| Sucrose Citrate Buffer | 600 | 4h | 2.0 kV, 400 Ω, 25μF | Yes | 0 |
| SHMG | 600 | 4h | 2.0 kV, 400 Ω, 25μF | Yes | 0 |
| 10% Glycerol and water* | 400 | Overnight | 1.5 kV, 800 Ω, 25μF | Yes | 9 cfu |
| 10% Glycerol and water (room temp)* | 400 | Overnight | 2.5 kV, 200 Ω, 25μF | Yes | 3 cfu |
| 10% Glycerol and water (room temp)* | 400 | Overnight  Addition of 2.5 % DMSO to recovery medium | 1.5 kV, 800 Ω, 25μF | Yes | 0 |
| 10% Glycerol and water (room temp)* | 400 | Overnight | 2.5 kV, 200 Ω, 25μF | Yes | 2 cfu |
| 10% Glycerol and water* | 400 (plasmid extracted from *G. vaginalis* ATCC 49145) | Overnight | 1.5 kV, 800 Ω, 25μF | Yes | 2 cfu |
| 10% Glycerol and water* | 400 (plasmid extracted from *G. vaginalis* ATCC 49145) | Overnight  Addition of 2.5 % DMSO to recovery medium | 1.5 kV, 800 Ω, 25μF | Yes | 2 cfu |

*Freshly made (not frozen) competent cells

**Supplemental Figure 1**: Performance of uppS and Sp^R^ gene SYBR green PCR assays. All reactions performed in duplicate using the calibrator plasmid as template. A) Standard curve for uppS assay, B) Standard curve for Sp^R^ gene assay, C) melt peak of the uppS PCR product, D) melt peak of the Sp^R^ gene product.

# Calibrator design

The following sequence shows the uppS gene fragment and the primer binding sites (JH0931 and JH0932 – highlighted in yellow) and the Sp^R^ gene fragment with primer binding sites (JH0949 and JH0950- highlighted in green). This 225 bp fragment was ligated into pIDTSmart Ampicillin (Supplemental Figure 2).

| Target | Primer name | Sequence |
| --- | --- | --- |
| uppS | JH0931  JH0932 | 5’-TTAGTGATCCTAGCCGCGTG-3’  5’-GCCGTCCATAATCACGCCTA-3’ |
| Sp^R^ gene | JH0949  JH0950 | 5’-TCAGGATGATGAAACCAACTCT-3’  5’-ACGAACTGCTAACAAAATTCTCTCC3-3’ |

**TTAGTGATCCTAGCCGCGTG**CCAGATTTTCCTAAAGGCAAAGTTCCTCGCCATGTAGG**CGTGATTATGGACGGCTCAGGATGATGAAACCAACTCT**ATATTAACTTTATGCCGTATGATTTTAACTATGGACACGGGTAAAATCATACCAAAAGATATTGCGGGAAATGCAGTGGCTGAATCTTCTCCATTAGAACATAG**GGAGAGAATTTTGTTAGCAGTTCGT**


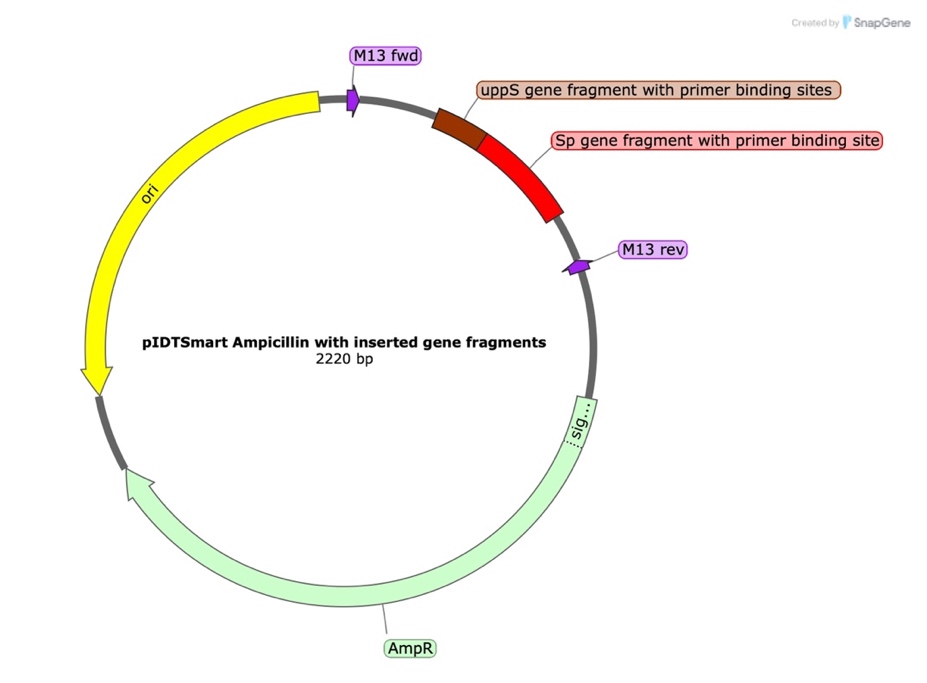


**Supplemental Figure 2**: pIDTSmart Ampicillin plasmid with inserted uppS gene fragment and primer binding sites and Sp^R^ gene fragment with primer binding sites.
